# Intensified wind pollination mediated by pollen dimorphism after range expansion in an ambophilous biennial *Aconitum gymnandrum*

**DOI:** 10.1002/ece3.2636

**Published:** 2016-12-20

**Authors:** Lin‐Lin Wang, Chan Zhang, Ming‐Liu Yang, Guo‐Peng Zhang, Zhi‐Qiang Zhang, Yong‐Ping Yang, Yuan‐Wen Duan

**Affiliations:** ^1^ Key Laboratory for Plant Diversity and Biogeography of East AsiaKunming Institute of BotanyChinese Academy of SciencesKunmingYunnanChina; ^2^ Plant Germplasm and Genomics Centerthe Germplasm Bank of Wild SpeciesKunming Institute of BotanyChinese Academy of SciencesKunmingYunnanChina; ^3^ Institute of Tibetan Plateau Research at KunmingKunming Institute of BotanyChinese Academy of SciencesKunmingYunnanChina; ^4^ College of Life SciencesHenan Normal UniversityXinxiangHenanChina; ^5^ College of Life ScienceYunnan Normal UniversityKunmingYunnanChina

**Keywords:** bumblebee pollination, marginal population, Qinghai–Tibet Plateau, refugium population, reproductive assurance, wind pollination

## Abstract

Pollination systems and associated floral traits generally differ between core and marginal populations of a species. However, such differences are rarely examined in plants with a mixed wind‐ and bumblebee‐pollination system, and the role of wind pollination during range expansion in ambophilous plants remains unclear. We compared floral traits and the contributions of bumblebee and wind pollination in refugium and marginal populations of the ambophilous plant *Aconitum gymnandrum*. We found that most floral traits differed between the two populations, and those traits associated with the shift to wind pollination were pronounced in the marginal population. Bumblebee visitation rates varied significantly, but were generally low in the marginal population. Wind pollination occurred in both populations, and the efficiency was lower than that of bumblebee pollination. Two types of pollen grains, namely round and fusiform pollen, were transported to a stigma by bumblebees and wind, but fusiform pollen contributed to wind pollination to a larger degree, especially in the marginal population. Our results suggest that wind pollination was enhanced by pollen dimorphism in the marginal population of *A. gymnandrum*, and wind pollination may provide reproductive assurance when bumblebee activity is unpredictable during range expansion, indicating that ambophily is stable in this species and shift in pollination system could be common when plants colonize new habitats.

## 1 Introduction

The geographic distribution of species is often the result of a complex history of range shifts, for example, the contraction or expansion of species distribution due to the glacial–interglacial cycles (Abbott & Brochmann, 2003). The current distribution of species at mid‐ to high latitudes could be attributed to range expansion from a “refugium” population at lower latitudes following global warming after the Pleistocene (Hewitt, 2000). Therefore, many extant plant species may have experienced range expansions and contractions in response to historical warming and cooling events (Svenning, Eiserhardt, Normand, Ordonez, & Sandel, 2015). After range expansion, marginal populations may be of smaller size and lower density than core populations owing to founder effects (Hardie & Hutchings, 2010) and the resultant vulnerability to environmental fluctuations (Frankham, 2005), and pollinator availability may be less reliable in marginal populations than that in core populations. Accordingly, under selective pressure from pollinator scarcity and unpredictability, the pollination system of marginal populations may differ substantially from that of core populations (Busch, 2005; Geber & Moeler, 2006; Herlihy & Eckert, 2005).

Uniparental reproduction, consisting of asexuality and selfing, could overcome low plant density and pollinator scarcity (Eckert, Samis, & Dart, 2006). Such a strategy may be an important evolutionary trend in marginal populations because it provides reproductive assurance during colonization and establishment at low density when outcrossing is limited (Barrett, 2002; Geber & Moeler, 2006; Schoen, Morgan, & Bataillon, 1996). However, reduced population genetic diversity owing to founder effects in marginal populations (Eckert, Samis, & Lougheed, 2008) would be further intensified after continuous uniparental reproduction, exposing populations to in the risk of extinction (Frankham, 2005). Therefore, selection for outcrossing would be favored in marginal populations, and wind pollination provides a solution to the conflict between small population size and maintenance of outcrossing in marginal populations. Wind pollination is considered to play an important role in conferring reproduction assurance under pollinator scarcity (Culley, Weller, & Sakai, 2002; Friedman & Barrett, 2009). For example, in the self‐incompatible and insect‐pollinated *Linanthus parviflorus*, pollination mediated by airborne pollen accounts for more than 60% of seed production from open‐pollinated flowers, providing one of the few examples supporting the hypothesis of reproductive assurance by wind pollination when pollinators are unreliable (Goodwillie, 1999).

Given that mechanisms of pollen dispersal differ between wind‐ and animal‐pollinated plants, the floral traits are also dissimilar. Generally, few ovules, unisexual flowers, reduced volatile emissions, and a high degree of sexual dimorphism in color and scent are often associated with the transition from animal pollination to wind pollination (Friedman & Barrett, 2008, 2009; Welsford, Hobbhahn, Midgley, & Johnson, 2016). Furthermore, other floral traits, such as reduced perianth and nonadhesive pollen, both of which could facilitate the pollen dispersal by wind, might also be expected in wind‐pollinated flowers. However, it remains unclear how these traits alter between core and marginal populations of plant species with a mixed animal‐ and wind‐pollination system (ambophily), especially for those species that have experienced a recent range expansion. In addition, wind‐ and animal‐mediated pollen dispersal may result in selection for different pollen size in plants with different pollination systems (Welsford et al., 2016), but whether pollen grains of ambophilous plants are morphologically differentiated as an adaptation to the different pollination modes requires further clarification.

The Qinghai–Tibet Plateau (QTP) is the largest and highest plateau in the world. Plants inhabiting the QTP are likely to have been strongly impacted by extinction, migration, or speciation during historical climatic oscillations, especially during the Quaternary (Liu, Duan, Hao, Ge, & Sun, 2014; Liu, Sun, Ge, Gao, & Qiu, 2012). Thus, the contemporary flora presents an opportunity to examine evolutionary shifts in pollination system in plants that have experienced range expansion on the QTP. *Aconitum gymnandrum* (Ranunculaceae) is a biennial herb native to the QTP with a mixed wind‐ and bumblebee‐pollination system (Duan, Zhang, He, & Liu, 2009; Zhang, Duan, & Liu, 2006). Phylogeographic analysis of the genetic structure of *A. gymnandrum* across its distribution range resolved two distinct lineages, generally congruent with eastern and western groups of populations, but evidence for range expansion from a refugium population after the Last Glacial Maximum was only identified in the eastern lineage based on haplotype diversity (Wang et al., 2009). Therefore, in the present research, we examined the evolutionary shift between wind and animal pollination and the possible changes in floral traits after range expansion by comparing the pollination systems of a refugium and a marginal population of *A. gymnandrum*. Specifically, we addressed the three following questions: (1) Do floral traits differ between the refugium and marginal population? (2) How does pollinator visitation differ between the refugium and marginal population? (3) How does the contribution of wind pollination to female fitness differ between the refugium and marginal population?

## 2 Materials and Methods

### 2.1 Study species and populations

**Figure 1:**
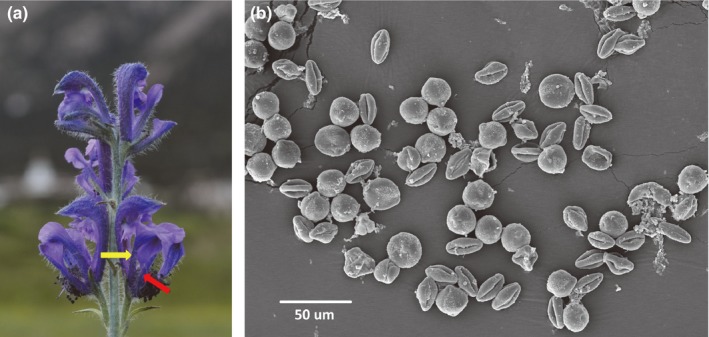
(a) Flowers of *Aconitum gymnandrum*, with a lower sepal and lateral sepal indicated by red and yellow arrows, respectively. (b) Two types of pollen grains of *Aconitum gymnandrum*, defined as round and fusiform pollen

*Aconitum gymnandrum* is the only species classified in *Aconitum* subg. *Gymnaconitum* (Ranunculaceae) (Li, 1988). The sepals and petals of *A. gymnandrum* are blue‐purple and showy, but the flowers exhibit several traits, that is, degenerate sepals and exposed anthers and stigmas (Figure 1a), which are unique in the genus *Aconitum* (Wang et al., 2001). Recently, the subgenus *Gymnaconitum* was raised to generic status (Wang, Liu, Yu, Gao, & Chen, 2013). *Aconitum gymnandrum* is widely but discontinuously distributed on the inner QTP and generally grows in degraded alpine grasslands and abandoned agricultural lands at altitudes ranging from 2230 to 4300 m a.s.l. (Wang et al., 2009). An individual plant consists of several branches, including one main terminal branch and multiple lateral branches. Each branch can be considered as an independent raceme. Flowers of *A. gymnandrum* are characterized by protandry and herkogamy (Zhang et al., 2006) and are pollinated by bumblebees and wind, but contribution of wind pollination to seed set is small in comparison with bumblebee pollination because of the high visitation rate and pollination efficiency of bumblebees (Duan et al., 2009).

On the basis of the genetic structure of *A. gymnandrum* (Wang et al., 2009), we selected a refugium population in Tongren County (35°29.7′ N, 102°16.5′ E; 3,190 m) and a northern marginal population at the Haibei Alpine Meadow Ecosystem Research Station (37°36.6′ N, 101°18.6′ E; 3,200 m) of the Chinese Academy of Science. The distance between the two populations is ca. 240 km, and both populations are situated in degraded alpine grasslands in Qinghai Province, but no grazing occurs in the two populations. Field experiments were carried out simultaneously at the two populations in June and July from 2012 to 2014.

### 2.2 Floral traits

Differences in floral traits between the refugium and marginal populations were investigated in 2012. We selected more than 50 plants, for each measured the plant height, inflorescence height, and number of flowers on the main branch, and calculated the flower density on the main branch. We also measured the maximum distance between the two lower sepals (Figure 1a) and the minimum width of the lateral sepals (Figure 1a) of flowers from randomly selected plants. In addition, 25 and 37 flower buds were sampled from different plants in the refugium and marginal populations, respectively, and fixed in FAA solution (formalin:acetic acid:100% ethanol, 5:5:90 v/v). All sampled flowers and buds were selected from the third flower or buds from the base of the main raceme to exclude a potential position effect on flower size and sex allocation (Zhao, Liu, & Conner, 2015; Zhao, Meng, Fan, & Du, 2008). In the laboratory, the number of carpels, ovules, and anthers was counted under a stereomicroscope. To quantify the number of pollen grains per flower, five nondehisced anthers per bud were squashed and diluted in 70% ethanol with a drop of detergent to obtain 5 ml suspension. The number of pollen grains was counted in 20 drops of the suspension (5 μl per drop) with a microscope, from which the total number of pollen grains per flower was calculated by multiplying the averaged pollen number of 20 replicates by 1,000. The pollen/ovule (P/O) ratio was then calculated. Pollen dimorphism, manifested as round and fusiform pollen grains, was determined in preliminary observations of *A. gymnandrum* pollen using a Hitachi (Tokyo, Japan) S‐4800 scanning electron microscope (SEM) (Figure 1b). Therefore, we counted the number of each type of pollen grain and calculated the percentage of fusiform pollen in each flower. In addition, using SEM micrographs with the scale bar as a reference, we measured the diameter (*D*) of round pollen and the long axis (*a*) and short axis (*b*) of the fusiform pollen from mixed anthers sampled from different flowers and fixed in FAA as described above. We calculated the size of round and fusiform pollen grains based on the surface area formula for a circle (1/4 × 3.14 × *D* ^2^) and ellipse (1/4 × 3.14 × *a *× *b*), respectively.

### 2.3 Bumblebee visitation and pollination efficiency

Pollinator observations and pollination efficiency were investigated from 2012 to 2014 in both populations on sunny days without strong wind. Bumblebees are the main animal pollinators of *A. gymnandrum* (Duan et al., 2009; Zhang et al., 2006). We treated all bumblebees as a single pollinator functional group without considering differences in bumblebee species. At the time of peak blooming in the populations, we labeled two to three inflorescences randomly and recorded the flowering stage and number of flowers on each inflorescence. Bumblebee visitation to these flowers was observed from 10:00 to 17:00. In total, we observed 950 flowers for 275 h in the refugium population and 270 flowers for 84 h in the marginal population in 2012 and 2014.

To determine the pollination efficiency of bumblebees, we emasculated and bagged flowers in the male phase in 2013 and 2014. When the stigma was receptive, we removed the bags and observed these flowers on sunny days. After each flower was visited once by a bumblebee, we re‐bagged the flower and fixed the stigma in FAA after 3 h. If no visitation was observed, the flower was discarded. In the laboratory, the number and type of pollen grains deposited on the stigma were determined under a stereomicroscope after the stigma was washed, rehydrated, softened in 8 N NaOH for 8 h, and stained with 1% aniline blue solution.

### 2.4 Airborne pollen and pollination efficiency

To examine the frequency of each pollen type in airborne pollen of the refugium and marginal populations, each slide covered with Vaseline on one side was affixed to a pole and placed vertically in both populations from 10:00 to 16:00 on sunny days from 2012 to 2014. All slides were put randomly in both populations with direction of the covered slide facing the prevailing wind, and the height of each slide was equal to the middle of the main raceme in each population. After collection, the slides were examined under a stereomicroscope, and the type and number of pollen grains affixed to the slide were determined.

To determine the potential and actual contributions to seed production by airborne pollen, 1,465 flowers from the third node of the inflorescence on different plants in the male phase were emasculated and covered with fine nylon nets of 1‐mm mesh in each study year. When the stigma had turned brown, which is an indicator of loss of stigma receptivity (Zhang et al., 2006), we collected the stigma from 771 flowers and fixed each in FAA. The type and number of pollen grains on each stigma were determined in the laboratory using the above‐mentioned methods. The remaining 694 flowers were collected before fruit dehiscence, and the number of seeds was counted.

### 2.5 Statistical analysis

Independent *t*‐tests were used to compare the floral traits between the refugium and marginal populations. A general linear model was employed with the aim of identifying differentiation in the pollination system of *A. gymnandrum* after range expansion from a glacial refugium population.

## 3 Results

### 3.1 Floral traits

Plants in the refugium population were taller and produced a greater number of flowers than plants in the marginal population, but the flower density per inflorescence was higher in the marginal population than in the refugium population (Table 1). No significant difference was observed in the distance between the two lower sepals, but the minimum width of the lateral sepals was markedly smaller in the marginal population than that in the refugium population (Table 1). The numbers of stigmas and ovules per flower were higher in the marginal population than those in the refugium population, but the opposite was true for the number of anthers and total number of pollen grains (Table 1). Thus, the P/O ratio was higher in the refugium population than in the marginal population. Importantly, pollen dimorphism was observed in both populations (Figure 1b). The number of round pollen grains was significantly higher in the refugium population than in the marginal population, but no significant difference was observed in the number and the percentage of fusiform pollen grains between the two populations. The size of round and fusiform pollen grains was 340.44 ± 15.71 μm^2^ (Mean ± *SE*,* N *=* *30) and 164.51 ± 3.70 μm^2^ (*N *=* *30), respectively. The difference in size between the two types of pollen grains was significant (*t *=* *14.23, *p *<* *.001).

**Table 1:** Floral traits (mean ± *SE*, with sample size in parentheses following the trait name) of the refugium and marginal population of *Aconitum gymnandrum* on the Qinghai–Tibet Plateau

Traits	Refugium pop.	Marginal pop.	*t*
Plant height (cm) (76)	93.23 ± 2.51	30.06 ± 0.98	26.22**
Flower number (20)	33.65 ± 2.33	16.33 ± 0.92	8.33**
Flower density per plant (56)	0.32 ± 0.01	0.54 ± 0.01	−13.05**
Distance of lower sepals (mm) (43)	9.04 ± 0.22	8.80 ± 0.21	0.81 ns
Min. width of lateral sepal (mm) (43)	2.11 ± 0.05	1.79 ± 0.04	4.85**
Anther number (25)	75.04 ± 1.60	63.95 ± 1.32	5.34**
Stigma number (25)	7.16 ± 0.33	8.7 ± 0.27	−3.59**
No. of round pollen (25)	1.11 ± 0.07 × 10^6^	0.88 ± 0.05 × 10^6^	3.73*
No. of fusiform pollen (25)	1.00 ± 0.34 × 10^5^	0.52 ± 0.11 × 10^5^	1.94 ns
No. of total pollen (25)	1.21 ± 0.06 × 10^6^	0.93 ± 0.05 × 10^6^	4.77**
Percentage of fusiform pollen (%) (25)	8.39 ± 2.64	6.02 ± 1.42	0.79 ns
No. of ovules (25)	90.56 ± 5.31	122.08 ± 5.12	−5.91**
P/O ratio (25)	1.43 ± 0.11 × 10^4^	0.79 ± 0.04 × 10^4^	8.44**

*p =* *.05; **p =* *.01; ns, nonsignificant.

### 3.2 Bumblebee visitation and pollination efficiency

**Figure 2:**
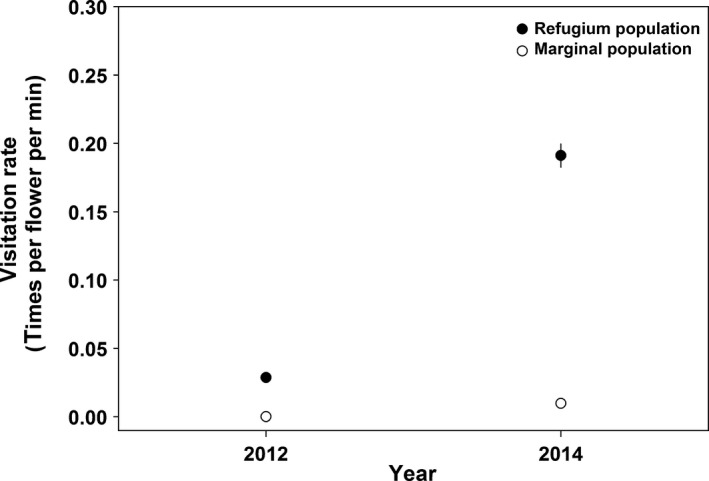
Visitation rates of bumblebees (mean ± *SE*) in the refugium (filled dots) and marginal (open dots) populations in 2012 and 2014

Visitation rates of bumblebees were affected significantly by year, population, and year × population interaction (Figure 2; Table 2). Specifically, visitation rates of bumblebees were higher in the refugium population than those in the marginal population in both years and were lower in 2012 than those in 2014 in both populations.

**Table 2:** Effects of population and year on visitation rate of bumblebees and seed number produced by airborne pollen in *Aconitum gymnandrum*

Source	Visitation rate of bumblebee				Seed number			
	Sum Squ.	*df*	*F*	*p*	Sum Squ.	*df*	*F*	*p*
Population	1.16	1	823.54	<.01	0.01	1	0.001	.98
Year	0.78	1	553.82	<.01	0.35	1	34.50	<.01
Population×year	0.61	1	436.03	<.01	0.01	1	0.53	.47

**Figure 3:**
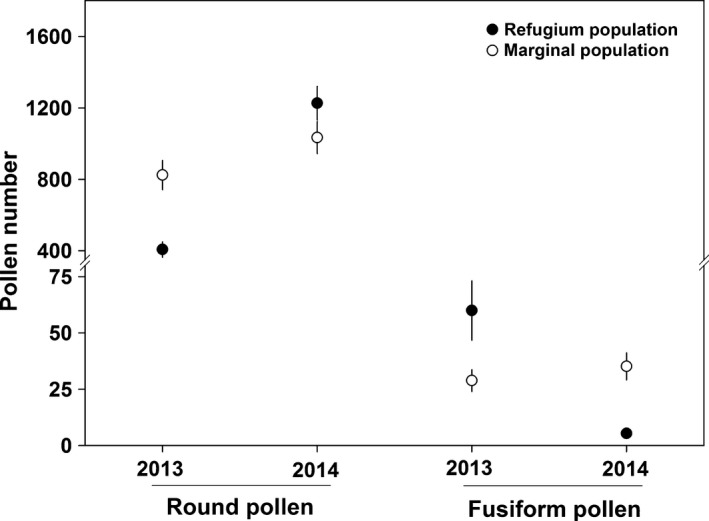
Number of round and fusiform pollen grains (mean ± *SE*) deposited on the stigma per flower after one visit by a bumblebee in the refugium (filled dots) and marginal (open dots) populations in 2013 and 2014. A break was employed in the vertical scale because of the great difference in the number of round and fusiform pollen grains

**Figure 4:**
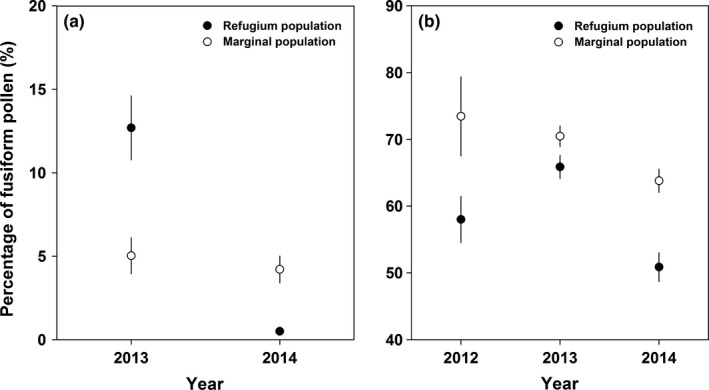
Percentage of fusiform pollen grains deposited on the stigma per flower after (a) one visit by a bumblebee (mean ± *SE*) in 2013 and 2014, and (b) by wind from 2012 to 2014 in the refugium (filled dots) and marginal (open dots) populations

After a single visit by a bumblebee, the number of pollen grains deposited on the stigma per flower was 922.76 ± 47.08 (Figure 3), and the number of round and fusiform pollen grains deposited differed significantly (Table 3). Importantly, the number of round pollen grains was significantly higher than that of fusiform grains, and the number of round and fusiform pollen grains varied significantly between the 2 years (Figure 3). The percentage of fusiform pollen grains was relatively stable in the marginal population between the 2 years, but varied significantly in the refugium population (Figure 4a; Table 4), although <20% fusiform pollen grains were deposited on the stigma by bumblebees in both populations in both years.

**Table 3:** Comparison of pollen number on the stigma per visit by bumblebees in 2013 and 2014, pollen number on the stigma of wind‐pollinated flowers in 2012–2014, and number of airborne pollen grains in 2012 and 2014 in the refugium and marginal populations of *Aconitum gymnandrum* on the Qinghai–Tibet Plateau. Pollen type and population were fixed factors, and year was a random factor

Source	Pollen number transported by bumblebee				Pollen number transported by wind				Number of airborne pollen grains			
	Mean Squ.	*df*	*F*	*p*	Mean Squ.	*df*	*F*	*p*	Mean Squ.	*df*	*F*	*p*
Pollen type	2.78	1	62.91	<.01	0.34	1	40.05	<.01	23.54	1	49.32	<.01
Population	0.01	1	0.23	.63	0.38	1	44.79	<.01	44.98	1	94.22	<.01
Pollen type × Population	0.01	1	0.24	.63	0.22	1	26.22	<.01	0.01	1	0.01	.97

**Table 4:** Comparison of percentages of fusiform pollen per flower on the stigma of flowers pollinated by bumblebees in 2013 and 2014 and by wind from 2012 to 2014 in *Aconitum gymnandrum*. Pollination type and population were fixed factors, and year was a random factor

Source	*df*	Mean Squ.	*F*	*p*
Pollination type	1	453723.62	788.69	<.01
Population	1	1438.04	2.50	.11
Pollination type × population	1	3033.08	5.27	.02

### 3.3 Airborne pollen and efficiency of wind pollination

**Figure 5:**
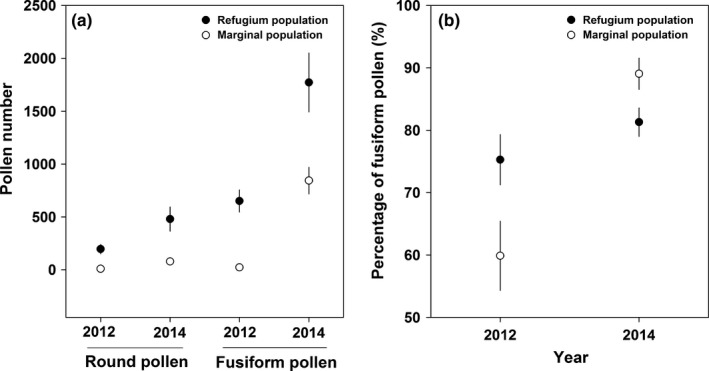
Number of airborne round and fusiform pollen grains (a) and percentage of fusiform pollen grains (b) captured by a slide covered with Vaseline (mean ± *SE*) in the refugium (filled dots) and marginal (open dots) populations in 2012 and 2014

The number of both round and fusiform pollen grains was higher in the refugium population than that in the marginal population, and the number of fusiform pollen grains was generally higher than that of round pollen grains (Figure 5a; Table 3). However, the percentage of fusiform pollen grains varied significantly between years and populations (Figure 5b).

**Figure 6:**
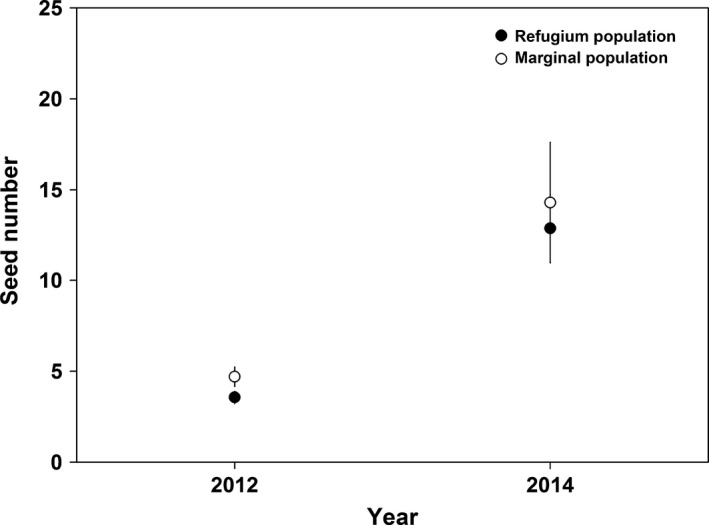
Seed number per emasculated and net‐covered flower (mean ± *SE*) in the refugium (filled dots) and marginal (open dots) populations in 2012 and 2014

The percentage of airborne fusiform pollen grains deposited on the stigma by wind was generally more than 50% (Figure 4b; Table 4). The percentage of fusiform pollen grains was generally higher in the marginal population than that in the refugium population across the 3 years (Table 4). The seed number resulting from flowers pollinated by airborne pollen grains varied significantly across years, but no significant difference between the two populations was observed (Figure 6, Table 2).

## 4 Discussion

### 4.1 Intensified wind pollination after range expansion

Evolution of a wind‐pollination system is generally associated with open habitats, unisexual flowers, dioecy, the uniovulate condition, small plain flowers, and a lack of nectar (Friedman & Barrett, 2008). Furthermore, a number of other species traits, including condensed inflorescences, flowers with exposed anther and stigma and great pollen production, are more common in wind‐pollinated plants than in animal‐pollinated plants (Culley et al., 2002; Weller, Sakai, Culley, Campbell, & Dunbar‐Wallis, 2006). However, a recent study suggests that uniovulate flowers and condensed inflorescences are conserved ancestral features in dioecious *Leucadendron* and might serve as an exaptation in transitions to wind pollination (Welsford et al., 2016). Nevertheless, it is generally accepted that floral traits differ markedly between wind‐ and animal‐pollinated plants, but the traits associated with the shift to wind pollination have been rarely examined at an intraspecific level. The present findings on the ambophilous *Aconitum gymnandrum* suggested that most of the measured floral traits differed significantly between the marginal and refugium populations. Importantly, condensed inflorescences, reduced width of the lateral sepals, and a higher number of stigmas in the marginal population were strongly suggestive of an evolutionary trend toward wind pollination after range expansion, because these traits facilitate pollen export and deposition by wind (Culley et al., 2002; Weller et al., 1998).

Reproductive assurance has been proposed to be an important selective pressure by maintaining outcrossing in unpredictable animal‐pollinator environments (Dafni & Dukas, 1986; Gomez & Zamora, 1996; Goodwillie, 1999; Karrenberg, Kollmann, & Edwards, 2002; Totland & Sottocornola, 2001). In geographically marginal populations, pollinator services are generally deemed to be less reliable than those of core populations because of the small population size and density due to founder effects (Aizen & Feinsinger, 1994; Groom, 1998). This assertion was supported by the present observations because the bumblebee visitation rate in the marginal population was considerably lower than that in the refugium population of *A. gymnandrum* (Figure 2). Furthermore, the bumblebee visitation rate was highly variable in both populations in different years (Table 2). Therefore, wind pollination may provide reproductive assurance and maintain outcrossing in the biennial *A. gymnandrum* when bumblebees are infrequently active in the marginal population, although the pollination efficiency of bumblebees is significantly higher than that of wind in this species (Duan et al., 2009).

The dispersal agents for pollen grains differ between wind‐ and animal‐pollinated plants; thus, the range in pollen grain size of wind‐pollinated plants (17–58 μm) tends to be smaller than that of animal‐pollinated plants (5–200 μm), although the difference in average pollen grain size is not significant among these groups (Wodehouse, 1935). In *A. gymnandrum*, two types of pollen grains that differ in morphology were observed, but the size of round and fusiform pollen grains was generally small (<30 μm) (Figure 1b). Examination of pollinated stigmas indicated that both types of pollen grains were transported by bumblebees and wind in both populations (Figures 3 and 5), but the ratio between the two pollen types was distinct. When flowers were visited once by a bumblebee, the proportion of fusiform pollen grains on the stigma per flower was <15%. However, the proportion of fusiform pollen grains on the stigma of wind‐pollinated flowers was more than 50% in the two populations across the 3 years. These results suggest that fusiform pollen grains could be dispersed more easily by wind than round pollen grains, and thus, we infer that wind pollination is mainly mediated by fusiform pollen in the ambophilous *A. gymnandrum*, although round pollen grains also contributed to wind pollination to a lesser degree and the percentage of fusiform pollen grains accounted for less than 10% of the pollen produced by a flower. Collectively, we conclude that wind pollination was enhanced in the marginal population of *A. gymnandrum*, which was generally mediated by fusiform pollen grains and facilitated by floral traits associated with wind pollination.

### 4.2 Ambophily in *Aconitum gymnandrum*

Most species in the tribe Delphinieae (Ranunculaceae) are exclusively adapted to bumblebee pollination (Bosch, Briones, Vergés, & Simon, 1997; Jabbour & Renner, 2012; Liao, Wang, Xie, Xiao, & Sun, 2007; Thostesen & Olesen, 1996). For example, in *Aconitum japonicum* var. *montanum*, the lateral sepals are considered to be important for successful pollination (Fukuda, Suzuki, & Murata, 2001), and the distance between the two sepals is under selection based on the body size of bumblebees (Brink, 1980). However, the sepals of *A. gymnandrum* are markedly degenerate in comparison with other *Aconitum* species, leading to the different floral architecture in *A. gymnandrum* (Wang et al., 2001, 2013). Specifically, the two lower sepals of *A. gymnandrum* occupy the position of the two lateral sepals of other *Aconitum* species. Thus, the distance between the two lower sepals in *A. gymnandrum*, rather than the distance between the two lateral sepals in other *Aconitum* species, might be under selection by bumblebees, which might be mirrored by the observation that the distance between the two sepals showed no difference between the two populations. The high pollination efficiency of bumblebees (Figure 2) in the present experiments strongly indicated that *A. gymnandrum* depends mainly on bumblebees for seed production, and the actual contribution to seed production by wind pollination was minor in comparison with bumblebee pollination, despite the stable occurrence of wind pollination in this species.

The frequency of ambophily is generally very low (Culley et al., 2002) but might be underestimated. For example, some plants that were once considered to be only wind pollinated or insect pollinated were identified to be pollinated by both wind and insect (Anderson, Overal, & Henderson, 1988; Gong et al., 2015; Peeters & Totland, 1999). Therefore, ambophily might represent an adaptation to different environments that vary in conditions favoring wind or animal pollination. Generally, for plants inhabiting open or alpine locations where animal pollinators are rare, wind pollination could be more common and dependable (Goodwillie, 1999; Totland & Sottocornola, 2001). In contrast, in closed habitats or low altitudes where animal pollinators are frequent, animal pollination could ensure reproductive assurance, and thus, ambophily is flexible and important to assure seed production. However, it is still uncertain whether ambophily is a stable stage or a transitional condition to either full wind pollination or animal pollination (Culley et al., 2002; Friedman & Barrett, 2009). Our observations of pollen dimorphism and the associated differentiation (to a certain degree) in pollen dispersal agents suggest that ambophily is a stable stage in *A. gymnandrum* because pollen dimorphism might be genetically controlled, which needs be demonstrated in future researches. Furthermore, selective pressures favoring floral traits associated with pollination by wind (e.g., degenerate sepals and pollen dimorphism) and by bumblebees (e.g., stable distance between the lower sepals) also support the hypothesis that ambophily is stable in *A. gymnandrum*. Collectively, wind pollination could overcome the shortage of bumblebee to assure seed production of this biennial in conditions (e.g., low temperature during glacial stage) or locations (e.g., new habitats) where bumblebee service is limited, and the mixed pollination system might be greatly beneficial for colonization of the high‐altitude Qinghai–Tibet Plateau by *A. gymnandrum* in comparison with other *Aconitum* species.

## Conflict of Interest

None declared.
